# Identification of a Homozygous Variant in the *CYP21A2* Gene by Next-Generation Sequencing Analysis of Circulating Cell-Free Fetal DNA

**DOI:** 10.3390/genes16030311

**Published:** 2025-03-05

**Authors:** Nadia Petrillo, Simone Marcella, Roberto Sirica, Monica Ianniello, Raffaella Ruggiero, Alessio Mori, Rosa Castiello, Cristina Ramiro, Rossana D’Angelo, Giuliano Pennacchio, Ermanno Barletta, Roberto Passaro, Antonio Fico, Giovanni Savarese

**Affiliations:** 1 AMES, Centro Polidiagnostico Strumentale s.r.l., Via Padre Carmine Fico 24, 80013 Casalnuovo Di Napoli, Italy; nadia.petrillo@centroames.it (N.P.); roberto.sirica@centroames.it (R.S.); monica.ianniello@centroames.it (M.I.); raffaella.ruggiero@centroames.it (R.R.); alessio.mori@centroames.it (A.M.); rosa.castiello@centroames.it (R.C.); cristina.ramiro@centroames.it (C.R.); rossanad83@icloud.com (R.D.); centroames@libero.it (A.F.); giovanni.savarese@centroames.it (G.S.); 2 CEINGE Biotecnologie Avanzate Franco Salvatore S.c.a.r.l., 80145 Naples, Italy; giuliano.pennacchio@yahoo.it; 3 Clinica Villa dei Fiori, Acerra, 80011 Naples, Italy; ermanno.barletta@gmail.com (E.B.); robertopassaromail@gmail.com (R.P.)

**Keywords:** CAH, NIPT, NGS, CYP21A2, CfDNA, hydrocortisone

## Abstract

Background/Objectives: Congenital adrenal hyperplasia (CAH) is an autosomal recessive disorder caused by mutations in the *CYP21A2* gene associated with 21-hydroxylase deficiency and increased levels of adrenal androgens. Affected females are at risk of ambiguous genitalia, while affected males show sexual precocity. Here, we present a case of a newborn female patient, characterized by ambiguous genitalia and previously identified as low risk for common aneuploidies by non-invasive prenatal testing (NIPT). Methods: We performed a NIPT, which showed a 46, XX genotype, confirmed by karyotype on the newborn’s DNA extracted lymphocytes. For clinical suspicion of CAH, we performed reverse dot blot and Multiple Ligation-dependent Probe Amplification (MLPA) of the *CYP21A2* gene on the patients and her parents’ DNA. Then, we performed on mother’s plasma NGS analysis with an in-house developed panel of genes for monogenic diseases, including the *CYP21A2* gene. Results: Reverse dot blot and MLPA detected the presence of the c.290-13A/C>G (I2 splice) mutation in heterozygosity in the parents and in homozygosity in the child, respectively. NGS detected the c.290-13A/C>G (I2splice) mutation in cell-free fetal DNA (cfDNA) in mother’s plasma with a variant allele frequency (VAF) of 67% with a fetal fraction (FF) of 5%. This latter suggests the presence of the variant both in the mother and in newborn cfDNA. Conclusions: The study reinforces the hypothesis that cfDNA can be used to identify point mutations, small insertions and/or deletions for the diagnosis of monogenic diseases, reducing the number of invasive tests and the risk of early miscarriages. Early detection of mutations in genes causing sexual development disorders could make it possible to start therapy in the womb.

## 1. Introduction

Congenital adrenal hyperplasia (CAH) is a group of autosomal recessive disorders due to mutations in *CYP21A2* gene leading to enzyme deficiencies involved in steroid hormone synthesis in adrenal glands [1]. Disease severity and phenotypic presentation depend on the location and extent of point mutations or deletions, which lead to complex allelic variations [2]. In 90–95% of CAH, defined as classical CAH, cases are characterized by steroid 21-hydroxylase (21-OH) deficiency that flows in atypical genitalia in female newborns and in sexual precocity in males [3]. Young women may present oligomenorrhea, polycystic ovaries, and hirsutism [4,5]. However, disease-related mortality has increased and therapeutic management remains challenging, with multiple long-term complications related to treatment and disease affecting growth, development, metabolic, cardiovascular conditions, and fertility [1]. Novel treatment approaches like hydrocortisone are emerging with the aim of mimicking physiological circadian cortisol rhythm and/or reducing adrenal hyperandrogenism independent of the suppressive effect of glucocorticoids [6]. For this purpose, it is necessary to diagnosis the disease as soon as possible to improve the life conditions of the CAH patients. Therefore, non-invasive prenatal testing (NIPT), using cell-free fetal DNA (cfDNA), firstly applied to monogenic diseases in 2012 [7], may help to provide an early diagnosis for single-gene diseases.

## 2. Case Report

**Figure 1:**
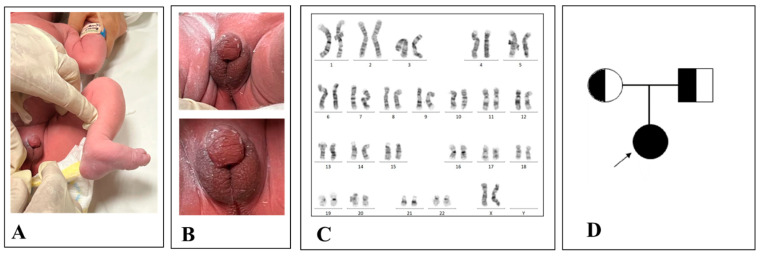
Patient’s ambiguous genitalia: congenital clubfoot (**A**) and hypospadic penis and empty scrotum (**B**) as per a virilized female. Patient’s genotype with a 46, XX genotype (**C**). Patient’s family tree (**D**).

We report a case of a female newborn patient characterized as low risk for common aneuploidies, investigated by NIPT. Structural ultrasound findings revealed a phenotypic female sex with clitoral hypertrophy. The patient at birth presented with a crooked foot (Figure 1A) and ambiguous genitalia (Figure 1B). Biochemical exams showed increased levels of adrenal hormones (Table 1). We hypothesized that the patient was affected by a severe form of classical CAH defined “salt-wasting CAH”.

This latter is usually associated with a worse prognosis due to low aldosterone production by adrenal glands leading to a high sodium loss by urines. If it is not diagnosed in time, symptoms appear within days or weeks after birth and, in some cases, death occurs [8]. Hence, we performed an analysis of the genotype derived from fetal lymphocytes and we found a 46, XX chromosomal asset confirming NIPT results (Figure 1C). In addition, we pieced the patient’s family tree identifying whether the patient and her parents were affected and not affected by the disease, respectively (Figure 1D).

**Figure 2:**
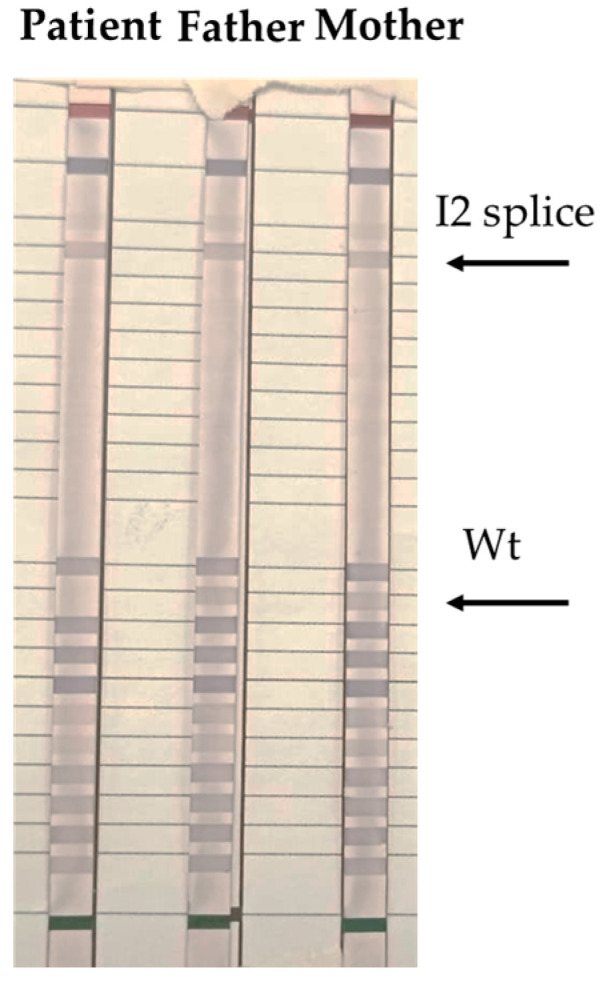
Arrows depict positions of bands obtained by reverse dot blot hybridization with the CAH Strip Assay, Vienna Lab regions of the *CYP21A2* gene whose mutations are amplified simultaneously by multiplex PCR with biotinylated primers: P30L, I2 splice, Del 8 bp E3 (G110del8nt), I172N, Cluster E6 (I236N, V237E, M239K), V281L, L307 frameshift (F306+T), Q318X, R356W, P453S, R483P. Wt, wild type.

**Figure 3:**
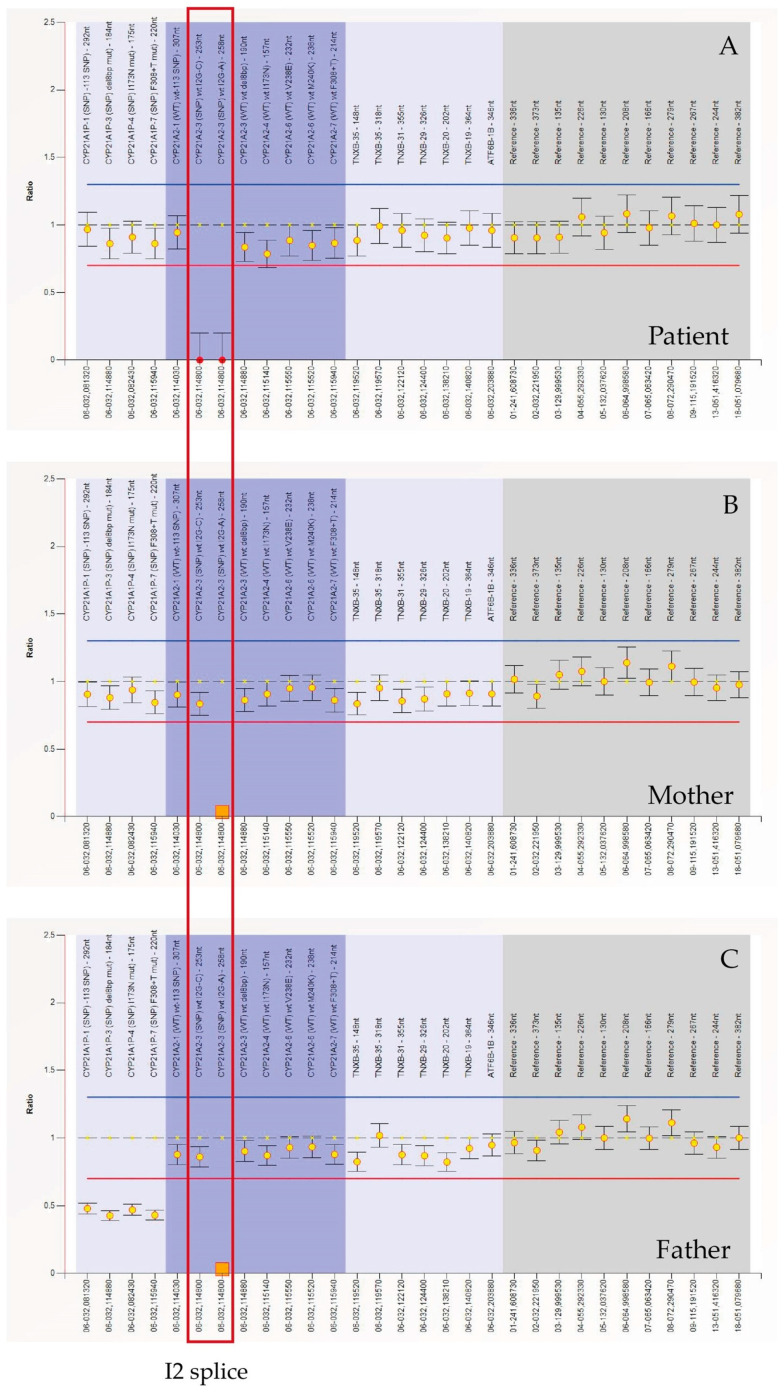
MLPA (Multiplex Ligation-dependent Probe Amplification, Probemix P050-C1 CAH, MRC-Holland) performed on patient’s, mother’s and father’s DNA. The orange square represents the c.290-13A/C>G (I2 splice) mutation of the *CYP21A2* gene in heterozygosity, of which both parents are carriers. The red dots represent the same mutation in homozygosity. MLPA (Multiplex Ligation-dependent Probe Amplification).

Then, to confirm clinical suspicion of the CAH disease, we performed a reverse dot blot (Figure 2) and Multiplex Ligation-dependent Probe Amplification (MLPA) (Figure 3) on *CYP21A2* gene of the patient and parental DNA. Therefore, we identified c.290-13A/C>G (I2 splice) mutation overlaps that results from the family tree analysis: homozygous for the patient and heterozygous for her parents. Specifically, for reverse dot blot, we used two panels for *CYP21A2* gene polymorphisms: a panel for wild-type (wt) polymorphism and a panel with 11 highest frequency polymorphisms inducing disease. After the PCR test, DNA fragments were hybridized with oligonucleotides probes and we found c.290-13A/C>G (I2 splice) mutation in both the patient and her parents. The patient was homozygous for c.290-13A/C>G (I2 splice) mutation, revealed also by the lack of signal in the wt stripe. In contrast, wt stripes were found in both father and mother confirming they were heterozygous for c.290-13A/C>G (I2 splice) mutation in *CYP21A2* gene.

MLPA was performed to detect the exact copy number of *CYP21A2* gene. We used MLPA Probemix P050-D1 CAH contain 30 MLPA probes with amplification products between 130 and 382 nucleotides. This includes eight probes for the *CYP21A2* gene and four probes for the *CYP21A1P* pseudogene. Figure 3 shows that CYP21A2 probes detected both wild-type sequences and c.290-13A/C>G (I2 splice) mutation in homozygosis (Figure 3A) and in heterozygosis (Figure 3B,C) in the patient and her parents, respectively. The following changes can be observed in the ratio chart as follows: the ratio for the specific probe targeting the mutated region drops to ~0.0 or a very low value. This occurs because the mutation disrupts the probe-binding site, preventing ligation and subsequent amplification. Other probes targeting unaffected regions of the *CYP21A2* gene maintain a normal ratio (~1.0). Different from their son, mother (B), and father (C) are heterozygous for c.290-13A/C>G (I2 splice) and the ratio is ~0.5 (indicating 50%of the expected signal).

Finally, we conducted a retrospective analysis performing next-generation sequencing (NGS) on circulating cfDNA. The NGS testing was performed on an aliquot of maternal plasma obtained during NIPT assay and stored in our Biobank at −80 °C to ensure the integrity of the DNA. This analysis employed a custom gene panel of 1069 genes including *CYP21A2* gene, and 100 copy number variations (CNVs) associated with monogenic diseases with significant clinical relevance and substantial impact on public health. The gene panel included a region of 3,490,181 base pairs and encompassed genes whose mutations (insertions/deletions, point mutations, duplications, de novo mutations, and rearrangements) were causative of a wide spectra of disorders (i.e., neuronal, skeletal, cardiac, intestinal, and kidney disorders) associated with an autosomal dominant, autosomal recessive and X-linked inheritance. Moreover, genes were chosen for their potential to enhance, during the prenatal screening, both the pregnancy management and early intervention after birth.

CfDNA and gDNA were amplified and repaired by using Kapa HyperPrep and KAPA hyperplus kits (Kapa Biosystems, Roche Diagnostics, Wilmingtonj, MA, USA), respectively, by means of End repair, A-Tailing, Ligate adapters, and Enrich DNA fragments to obtain the final sample for NGS. According to the KAPA HyperPrep kits and given the fragmentation of cfDNA, no additional fragmentation steps were required for the sample extracted from maternal plasma. In contrast, according to KAPA HyperPlus kits manufacturer’s instructions, first of all, gDNA was fragmented to achieve the size required by the applied Illumina short-reads sequencing technology. DNA quantification was performed using a Qubit 3.0 Fluorometer with the Qubit ds- DNA HS (High Sensitivity) Assay Kit fluorescent dye method. Sequencing was carried out on NovaSeq 6000 (Illumina Inc., San Diego, CA, USA) to a mean sequencing depth of at least 600X. Bioinformatic analysis were conducted taking advantage of in-house pipelines.

**Figure 4:**
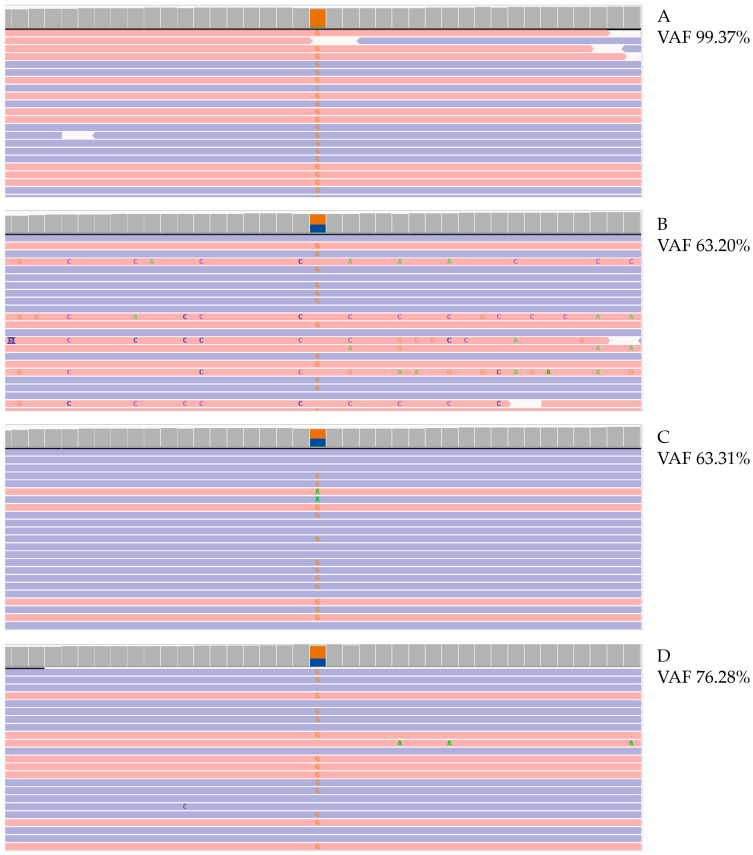
Molecular analysis of *CYP21A2* gene. Photographs of Integrative Genomics View (IGV) representation of the c.290-13A/C>G (I2splice) mutation in *CYP21A2* gene identified with NGS, VAF calculated on affected patient (**A**), patient’s father (**B**), patient’s mother (**C**), and cfDNA (**D**). VAF, variant allele frequency. Red and blue lines represent the aligned reads in paired-end. Orange squares represent the frequency of reads carrying alternate allele.

Finally, NGS analysis of parental and fetal DNA revealed c.290-13 A>C/G (I2 splice) mutation in *CYP21A2* gene with a variant allele frequency (VAF) of 67% and FF of 5% in newborn and of 63% in both parents (Figure 4D).

However, these data suggest the presence of the variant both in the mother and in cfDNA, leading to the conclusion that fetal DNA can be used for the identification of point mutations and small insertions and/or deletions for the diagnosis of monogenic diseases, potentially reducing the number of invasive tests and the risk of early miscarriage [9].

## 3. Discussion

The lack of the enzyme 21-OH is the most common cause of CAH due to *CYP21A2* gene mutations leading to reduced cortisol production that flows into increased ACTH production and consequentially an increase in androgen output from the adrenal glands. An excess of fetal adrenal androgens interferes with the development of the female external genitalia in utero, resulting in ambiguous genitalia [3]. In fact, females are virilized at birth because their external genitalia, including the penile urethra and fused labioscrotal folds, resemble males [10]. By contrast, the male newborns may not have received a diagnosis because they do not present with life-threatening symptoms, and do not have genital ambiguity to warn doctors but are presented with a salt loss emergency and may be characterized by impaired fertility [11]. Therefore, a standard CAH screening may be useful to perform an early diagnosis of disease mainly for females. Interestingly, *CYP21A2* gene is located on the short arm of chromosome 6 near to HLA locus [12], a chromosomal region, known to be subject to frequent mutations among the different classes of HLA [13]. In most countries, CAH diagnosis is mainly performed after birth by biochemical analysis in particular by 17-OHP assay that in the severe forms of the disease is very high [14]. The 17-OHP can become normal after a stimulus test with the Synacthen test, with the commercial name of ACTH. Specifically, 0.5 mg of ACTH is injected intramuscularly, then, after an hour, the basal 17-OHP dosage is performed and if it exceeds 1000 nanograms per deciliter the test is considered indicative of pathology [15]. Therefore, we explored a case of a female newborn affected by classical CAH and characterized by external ambiguous genitalia and high androgen levels. Firstly, we identified c.290-13A/C>G (I2 splice) mutation on *CYP21A2* gene by PCR and reverse dot blot in homozygosis and in heterozygosis in newborns and in parents, respectively. The point mutation leads to an intronic variant resulting in a splicing mutation that occurs in intron region 2, 13pb upstream of exon 3 [16]. Altered splicing flows in abnormal mRNA which may either retain intronic sequences or induce exon 3 skipping [17]. Because exon 3 encodes a critical segment of the 21-OH enzyme, altered splicing generates a stop codon, leading to either a truncated, non-functional protein or mRNA degradation via nonsense-mediated decay [18]. Therefore, the outcome is a partial or complete loss of enzyme activity. The same mutation was confirmed by MLPA analysis and NGS. The main goal of this case is to highlight the importance of prenatal screening to give a definitive and early diagnosis of monogenic disorders. Katthab and co-workers identified 14 pregnants whose fetuses were at high risk for CAH. The authors demonstrated that fetuses’ allelic compositions could be responsible abnormalities in *CYP21A2* gene. They demonstrated that the results obtained from non-invasive procedures were comparable to invasive CAH diagnostic procedures 100% of the time [19]. In the same way, Ma et al. collected 39 plasma samples from pregnants whose gestations ranged from the first to the second trimester and performed NGS on maternal and paternal DNA to identify whether the fetuses inherited wt or pathological CYP21A2 alleles. They showed that CAH could be identified in all 14 participants, with an early diagnosis at eight weeks of gestation proving that testing maternal plasma can be an alternative to invasive procedures [20]. New et al. used massively parallel sequencing to demonstrate the utility of cfDNA testing and the strategy for prenatal diagnosis of CAH in affected families. Sequence analysis was performed using mutation linked to the *CYP21A2* gene in parents. In 14 families affected by CAH due to CYP21A2 pathogenic variants, seven affected fetuses, five carriers, and two unaffected fetuses were identified as early as five weeks and six days of gestation [21]. However, even if non-invasive methods previously described using cfDNA can be helpful for prenatal screening, there is still much work to do to replace the gold standard of invasive tests like amniocentesis and villocentesis. In fact, for the implementation of the cfDNA-based test as a diagnostic test for prenatal determination of fetal inheritance of monogenic disorders to be successful, some factors should be considered: low FF (<4%) [22], multiple pregnancies [23], vanishing tween presence [24], maternal somatic mosaicism [25], maternal transplantation [26] or maternal DNA contamination [27]. While the use of cfDNA for the prenatal diagnosis of CAH offers a non-invasive alternative to traditional methods, several limitations must be considered to ensure accurate and reliable results.

Maternal DNA contamination is one of the primary challenges in cfDNA analysis because cfDNA is mixed with a substantial proportion of maternal cfDNA in maternal plasma and to distinguish fetal-specific genetic variants from maternal background noise can be difficult [28]. Maternal DNA contamination may lead to false-negative results whether it masks fetal mutations or false-positive findings whether maternal mutations are incorrectly attributed to the fetus [29]. Then, although sequencing technologies have improved substantially, technical errors and bioinformatics challenges persist. Therefore, false positives may arise from sequencing artifacts, while false negatives can result from low read depth or allelic dropout. Rigorous quality control measures and validation studies are crucial to improving the robustness of cfDNA-based CAH diagnosis [30]. In contrast, advanced bioinformatics techniques and targeted sequencing approaches are required to overcome this problem. In fact, the advanced computational algorithms help to isolate the cfDNA contribution from maternal DNA, improving diagnostic accuracy [31]. Thanks to these advancements, non-invasive prenatal diagnosis of CAH is now much more accurate and safe, reducing the risk of errors due to maternal DNA contamination. Another limitation is the potential presence of mosaicism, where different cells in the fetus may have different genetic compositions [32]. If CAH mutation is present in only a subset of fetal cells, cfDNA analysis might not accurately detect the full extent of the genetic variation [33]. This can lead to discrepancies between cfDNA findings and actual fetal genotype, complicating diagnostic conclusions. Further validation through invasive diagnostic methods such as amniocentesis may be necessary in cases where mosaicism is suspected.

The interpretation of VAF in cfDNA-based diagnosis presents additional difficulties. Since cfDNA originates from both the fetus and the mother, the proportion of mutant alleles detected does not always correlate directly with zygosity or disease severity. Variability in FF, affected by gestational age, maternal body mass index [34], and placental health [35] can further complicate VAF interpretation. Low FF may reduce the sensitivity of cfDNA-based detection, leading to ambiguous results that necessitate confirmation tests [36,37]. Despite its advantages, cfDNA-based prenatal diagnosis of CAH faces significant limitations that must be acknowledged and addressed. Maternal DNA contamination, potential fetal mosaicism, and complexities in VAF interpretation highlight the need for cautious interpretation of cfDNA findings. Ongoing advancements in sequencing technologies, bioinformatics pipelines, and FF enrichment strategies may help overcome these obstacles, but until then, cfDNA should be used in conjunction with traditional diagnostic approaches to ensure accuracy and reliability.

Moreover, large-scale studies are necessary for the prenatal diagnosis of CAH to improve the accuracy, reliability, and clinical utility of current testing methods. Given the complexity of cfDNA analysis, the potential for mosaicism, and the influence of maternal DNA contamination, further research is essential to refine diagnostic protocols, reduce false positives/negatives, and establish standardized guidelines for NIPT of CAH.

The patient had a low risk for aneuploidies and then, to evaluate whether the mutation was present on cfDNA, we performed a retrospective analysis on the parental DNA stored in Biobank, on cfDNA of the patient and we identified an FF of 5% and a VAF of 67%. These data suggest that the variant was present in both parents and the fetus, indicating the potential for early diagnosis of CAH. However, early diagnosis has historically been challenging for several reasons. One key difficulty is that CYP21A1P is a non-functional pseudogene that is not translated [14]. Non-homologous recombination between the functional *CYP21A2* gene and the pseudogene can occur, resulting in 1–2% of the mutations in *CYP21A2* gene being due to gene conversion events rather than direct inheritance from parents [38]. This complicates the analysis, as NGS can amplify both CYP21A2 and the highly similar CYP21A1P, making it difficult to distinguish them. In some cases, to address this issue, a primary PCR is performed that specifically amplifies all exons of the *CYP21A2* gene while excluding CYP21A1P. This product of amplification undergoes sequencing, but also to smaller PCRs and is therefore sequenced with Sanger Sequencing. The product of the initial PCR is further analyzed using enzymatic digestion, which allows differentiation between non-deleted and deleted alleles. It is crucial that the polymerase enzyme used in these polymerase chain reactions has proofreading activity. This ensures that the enzyme can self-correct during amplification, thereby increasing the accuracy of the PCR process. These technical challenges contribute to the difficulty of achieving early diagnosis of CAH. However, in our case study, NGS was able to detect pathogenetic variants. In fact, PCR is usually performed when VAF is <50% whereas mutations seen in heterozygosis and in homozygosis in the parents and the daughter were 63% and 67%, respectively. In our study, the deviation from the expected 50% allele frequency in a homogeneous DNA sample is interpreted in the context of a mixed cfDNA sample, where maternal DNA predominates and the fetal fraction constitutes only about 5% of the total DNA. In such samples, a heterozygous variant present exclusively in the fetal DNA would theoretically result in a VAF of approximately 2.5% (i.e., half of the fetal fraction) rather than the classical 50% observed in pure genomic DNA. Consequently, an observed VAF of 67% indicates that the mutant allele is not evenly distributed across all DNA sources but is instead enriched within the minor component of the sample.

This allelic imbalance enables an assessment of whether the variant is present in a heterozygous or homozygous state. However, this evaluation is inherently limited by the low FF, as only a VAF deviation ranging from about half of the FF to levels suggestive of mutation dominance can raise suspicion for a mutation. Our approach is deliberately conservative to minimize false negatives and ensure that potential mutations are not overlooked, even though this may lead to an increased number of false positives.

To enhance the accuracy of variant detection under these challenging conditions, we have employed a deep sequencing strategy with an average coverage of 600X. This high read depth is crucial for reliably detecting low-frequency variants in cfDNA and ensures that even those variants present at very low levels are not overlooked. Furthermore, any suspected mutations are confirmed using orthogonal methods validated specifically on cfDNA rather than on conventional genomic DNA.

Furthermore, this underlines that NGS is able to identify a mutation in *CYP21A2* gene. In addition, reverse dot blot and MLPA were also able to identify mutations only in *CYP21A2* gene avoiding pseudogene involvement. The main goal of our study was to pave the way to perform an early diagnosis starting from cfDNA and preventing corrective actions after birth. Early diagnosis would also enable prenatal treatment with drugs to manage adrenal pathways, ultimately improving the quality of life for individuals affected by CAH. To date, there are different therapies for CAH treatment. Specifically, hydrocortisone reduces the stimulation of the androgens pathway preventing further virilization and following normal growth and development [39]. The usual requirement of hydrocortisone for the treatment of a classical 21-OH form of CAH is about 10-15 mg/m^2^/day divided into two or three doses per day and for the non-classical 21-OH 5-8 mg/m^2^/day divided into two or three doses per day [40,41]. New treatment strategies are being developed to either replicate the physiological circadian rhythm of cortisol or to reduce adrenal hyperandrogenism without relying on the suppressive effects of glucocorticoids such as hydrocortisone [18]. In fact, as reported in Table 1, hydrocortisone reduced the patient’s biochemical parameters. In conclusion, NIPT for monogenic disorders enables the early detection of pathogenic mutations, potentially allowing therapeutic intervention during pregnancy. Furthermore, cfDNA analysis holds promise not only for enhancing the quality of life for patients with CAH but also for improving the conditions of patients affected by other genetic disorders.

## 4. Conclusions

In summary, cfDNA analysis by NGS may be useful to perform and early diagnosis of CAH as well as for other monogenic disorders. It is important that patients with a screen-positive result for a genetic anomaly are counseled appropriately and advised to undergo an invasive diagnostic procedure for confirmation before any decisions regarding pregnancy outcome.

## Figures and Tables

**Table 1:** Patient’s adrenal hormone levels of five different dates. The measurements include 17-OHP, ACTH, testosterone, DHEA-S, androstenedione, cortisol, and renin. The data illustrate a significant reduction in hormone levels over time, reflecting the patient’s response to treatment. 17-OHP, 17 hydroxy-progesterone; ACTH, adrenocorticotropic hormone; DHEA-S, dehydroepiandrosterone sulfate.

	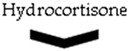					
Hormone Profile	1st Month	2nd Month	3rd Month	4th Month	5th Month	Normal Range Values
17-OHP (ng/mL)	>20	208	4.17	0.2	0.43	0.2–2, 9
ACTH (pg/mL)	724	549	22.5	133.1	9.6	7.0–65
Testosterone (ng/dL)	389.9	275.9	7.8	0.5	0.05	Male < 10 years:0.30 Female < 10: 0.01–0.12
DHEA-S (mcg/dL)	2986	1600	144	798	261	<2500
Androstenedione (ng/mL)	>10	86.4	1.75	30	72	20–290
Cortisol (mcg/dL)	12.72	13.63	<0.5	2.9	22.7	Morning: 3.2–12.0 Evening: 0.1–8.0
Renin (pUI/mL)	88.23	608.30	149.2	55	26.2	3.11–41.2

## Data Availability

The data presented in this study are available on request from the corresponding author. The data are not publicly available due to privacy restrictions.
